# A Phase II, Double-Blind, Randomized, Placebo-Controlled, Multicenter Study Evaluating the Efficacy and Safety of Alpha-1 Antitrypsin (AAT) (Glassia^®^) in the Treatment of Recent-Onset Type 1 Diabetes

**DOI:** 10.3390/ijms20236032

**Published:** 2019-11-29

**Authors:** Yael Lebenthal, Avivit Brener, Eli Hershkovitz, Naim Shehadeh, Shlomit Shalitin, Eli C. Lewis, Dana Elias, Alon Haim, Galia Barash, Neta Loewenthal, Nehama Zuckerman-Levin, Michal Stein, Naveh Tov, Marianna Rachmiel

**Affiliations:** 1 The Jesse Z. and Sara Lea Shafer Institute for Endocrinology and Diabetes, National Center for Childhood Diabetes, Schneider Children’s Medical Center, Petah-Tikva affiliated with Sackler School of Medicine, Tel Aviv University, Tel Aviv 6997801, Israel; avivit.brener@gmail.com (A.B.); shalitin@netvision.net.il (S.S.); 2 Pediatric Diabetes Unit, Soroka Medical Center, Beer-Sheva affiliated with Faculty of Health Sciences, Ben-Gurion University of the Negev, Beer-Sheva 8410501, Israel; elih@bgu.ac.il (E.H.); alonhaim@bgu.ac.il (A.H.);; 3 Pediatric Diabetes Unit, Meyer Children’s Hospital, Rambam Medical Center, Haifa affiliated with Bruce Rappaport Faculty of Medicine, Technion, Haifa 319601, Israel; n_shehadeh@rambam.health.gov.il (N.S.); nzuckerln@gmail.com (N.Z.-L.); 4 Department of Clinical Biochemistry and Pharmacology, Faculty of Health Sciences, Ben Gurion University of the Negev, Beer-Sheva 8410501, Israel; eli.c.lewis@gmail.com; 5 Kamada Ltd., 2 Holzman St., Science Park, Rehovot 7670402, Israel; danafire50@gmail.com (D.E.); michals@kamada.com (M.S.); naveht@kamada.com (N.T.); 6 Pediatric Endocrinology Unit, Assaf Harofeh Medical Center, Zerifin, Israel affiliated with Sackler School of Medicine, Tel Aviv University, Tel Aviv 6997801, Israel; shakshuka2@hotmail.com (G.B.); rmarianna@gmail.com (M.R.)

**Keywords:** alpha-1 antitrypsin, type 1 diabetes, children and adolescents, beta cell preservation

## Abstract

Our aim was to assess the efficacy, safety, and tolerability of alpha-1 antitrypsin (AAT) as a therapeutic modality for β-cell preservation in patients with recent-onset type 1 diabetes. Seventy type 1 diabetes patients (37 males; mean age 13.1 ± 4.1years) were randomized to treatment with 22 infusions of AAT (Glassia^®^) (60 or 120 mg/kg) or placebo. The primary outcome was the area under the curve (AUC) of C-peptide from a 2-h mixed-meal tolerance test after 52 weeks. At week 52, C-peptide was 0.9, 0.45, and 0.48 pmol/mL in the AAT-120, AAT-60, and placebo groups (*p* = 0.170 and *p* = 0.866 vs. placebo, respectively). The declines in C-peptide glycated hemoglobin (HbA1c) and the total insulin dose (U/kg) were similar across groups. Within the predefined 12–18-years subgroup, the C-peptide AUC decreased significantly in the placebo and AAT-60 groups (−0.34 and −0.54 pmol/mL, respectively, *p* < 0.01), with a borderline decrease in the AAT-120 group (−0.29 pmol/mL, *p* = 0.047). The mean HbA1c level was significantly lower in the AAT-120 group compared to the placebo (6.7% ± 0.9% vs. 8.2 ± 1.4%, *p* = 0.05), and a higher percentage of patients attained HbA1c ≤ 7% (75% vs. 25%, *p* = 0.05). AAT was tolerated well, with a similar safety profile between groups. The AAT intervention showed promise in the subgroup of adolescents with recent-onset type 1 diabetes. Further studies are warranted to determine the impact and proposed mechanism of action of AAT in β-cell preservation.

## 1. Introduction

Type 1 diabetes (T1D) is a chronic autoimmune disease that results in the loss of insulin-secreting β-cells. The progression of severe insulin deficiency leads to chronic hyperglycemia and, if not appropriately treated, to potential complications [1]. There has been accumulating evidence on the role of the innate arm of the immune system in driving the autoinflammatory milieu, which subsequently drives and facilitates T-cell-mediated autoimmunity and provides antitolerogenic signals [2,3]. Inflammatory restraint, as well as the modulation of immunity toward β-cells, are sought in order to attenuate β-cell destruction and promote the maintenance of residual insulin secretion.

Alpha-1 antitrypsin (AAT) is an intrinsic protein with immunomodulatory properties. Its levels increase during acute phase responses due to the presence of interleukin-1 (IL-1)- and IL-6-responsive elements inside the promoter region of the AAT gene [4]. AAT functions in part as a serine-protease inhibitor that targets several inflammatory enzymes that are all released during cell injury and that are involved in the upstream activation of inflammation [5]. Important proinflammatory mediators, including IL-1β and TNFα, which are highly involved in T1D, are inhibited by AAT at multiple levels [6].

Although cellular targets of AAT include primarily innate immune cells, treatment with AAT promotes regulatory T-cell (Treg) differentiation and expansion in various conditions [7,8,9,10,11,12]. AAT treatment of nonobese diabetic mice was found to postpone the onset of spontaneous diabetes, enable the preservation of islet architecture [13,14], and provide a cytoprotective environment after allogeneic islet cell transplantation [15,16]. These findings have shown promise and led to phase I/II trials of AAT as a therapeutic modality for β-cell preservation in recent-onset T1D patients, demonstrating a high safety profile [17,18,19,20]. Gottlieb et al. reported on an 8-week course of AAT therapy at a dose of 80 mg/kg body weight once per week for 8 weeks in 12 adult T1D patients [18]. AAT intervention was tolerated well and was partially beneficial to β-cell function: therapy with AAT either increased C-peptide responses or inhibited the decline in C-peptide responses in some of the patients. The Research Trial of Aralast in New Onset Diabetes (RETAIN) study group reported that 12 infusions of human plasma-derived AAT (Aralast NP, Baxalta, Inc.) at a dose of 45–90 mg/kg/dose over 18 weeks were found to be safe in new-onset T1D patients aged 8–35 years [17]. We previously reported that 18 infusions of AAT (Glassia^®^, Kamada 48 Ltd.) over 28 weeks were safe and tolerated well in pediatric subjects with recently diagnosed T1D [19]. Our prospective open-label phase I/II extension study demonstrated that the administration of multiple repeated AAT infusions (up to 36) to AAT-sufficient pediatric T1D patients from diabetes onset revealed normal growth and pubertal progression through adolescence to the attainment of full puberty and near-adult height [20].

The current study was designed to assess the effects of AAT on pancreatic β-cell reserves in patients with recent-onset type 1 diabetes.

## 2. Results

**Figure 1:**
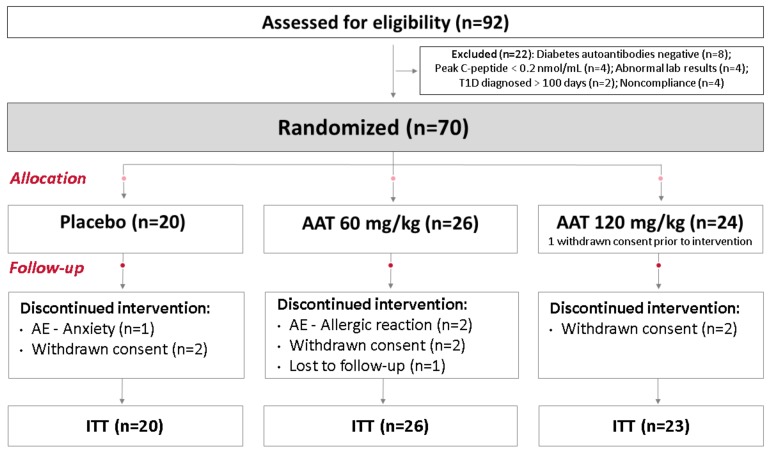
CONSORT diagram: 92 patients were assessed for eligibility: 22 did not meet the inclusion criteria and were excluded due to negative diabetes autoantibodies, no evidence of β-cell reserves, abnormal labs, type 1 diabetes having been diagnosed over 100 days ago, or noncompliance. Seventy participants were randomized and allocated into one of three groups: placebo or 60 or 120 mg/kg/dose of AAT. One participant allocated to the AAT-120 group withdrew consent prior to the intervention. Therefore, the intention-to-treat (ITT) analysis included 69 participants.

Ninety-two subjects were screened: seventy subjects met the inclusion criteria and were randomized to either the AAT-60 mg/kg, AAT-120 mg/kg, or placebo group (one participant allocated to the AAT-120 mg/kg group withdrew consent prior to the intervention). Thus, the ITT analysis included 69 participants (Consolidated Standards of Reporting Trials CONSORT diagram in Figure 1). The baseline characteristics upon randomization of these three groups were similar (Table 1).

Thirty-seven participants (54%) were males, the mean age of participants was 13 years, and there were no significant differences between groups in terms of sex, age, or BMI. Thirty-one participants (45%) presented with diabetic ketoacidosis (DKA) at the time of diagnosis. The time elapsed from diabetes onset to the first dose of AAT was, on average, 71 days. Mean HbA1c at baseline was 8.5% (69 mmol/mol), and peak C-peptide was 0.63 pmol/mL. While 94% of participants were positive for anti-GAD, 48% were positive for both pancreatic autoantibodies.

### 2.1. ITT Analysis

**Figure 2:**
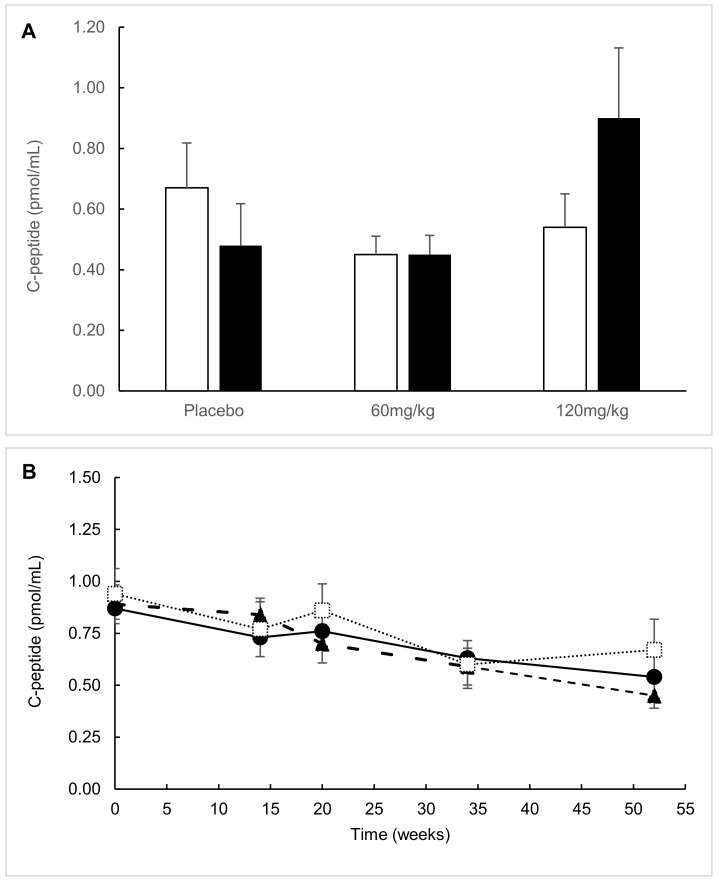

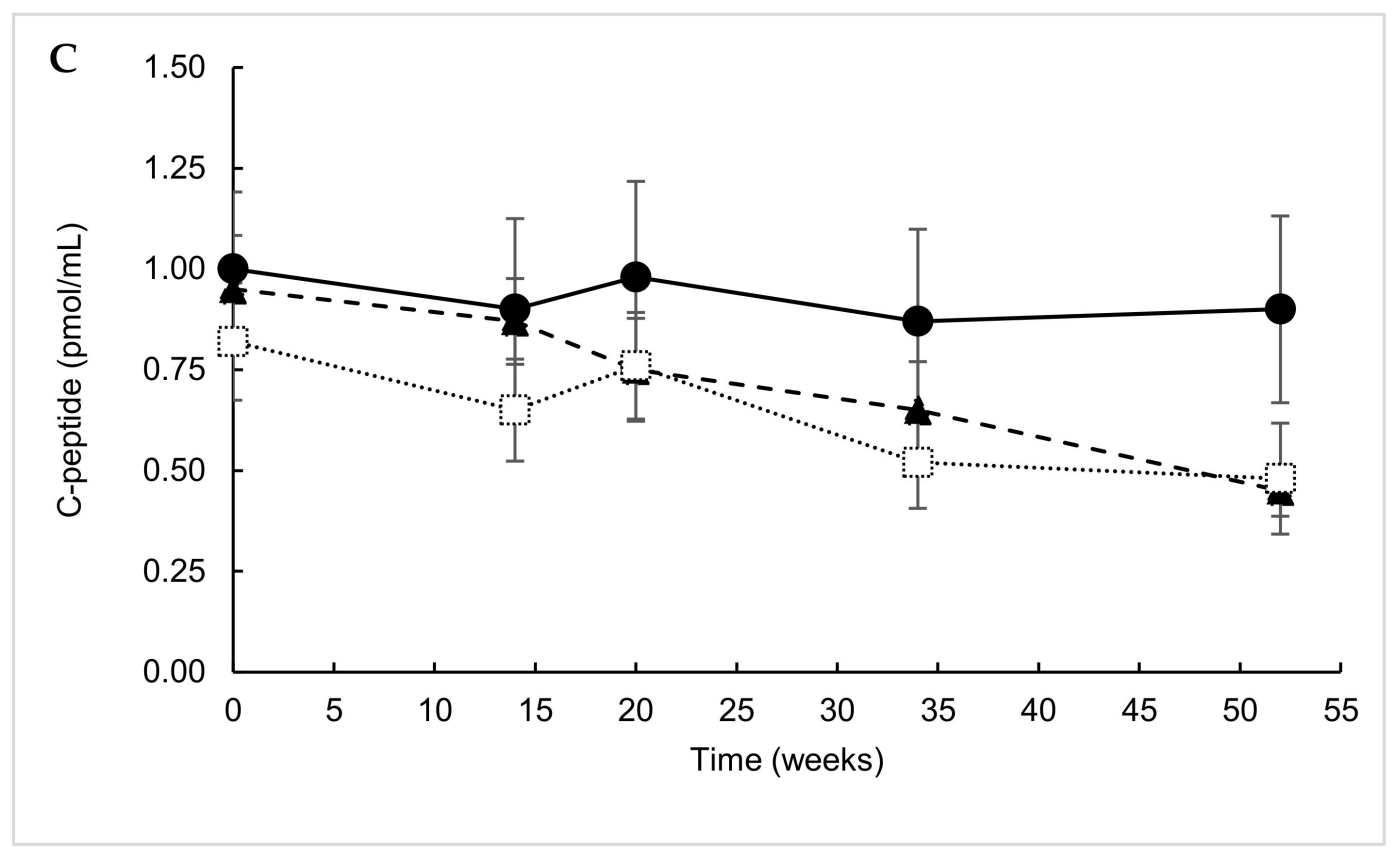
C-peptide area under the curve (AUC) stratified by treatment group. (**A**) C-peptide AUC at the study’s end (52 weeks) in the intention-to-treat (ITT) population versus the 12–18-year subgroup. White bars = ITT analysis and black bars = 12–18-year subgroup. (**B**) C-peptide AUC during the study in the ITT population. Dotted line = placebo group, dash–dot line = AAT-60 mg/kg group, and solid line = AAT-120 mg/kg group. (**C**) C-peptide AUC during the study in the 12–18-year subgroup. Dotted line = placebo group, dash–dot line = AAT-60 mg/kg group, and solid line = AAT-120 mg/kg group.

Fifty-two weeks after treatment initiation, various extents of decline in MMTT-stimulated C-peptide secretion were observed between study participants (Figure 2). In the ITT analysis, C-peptide AUC decreased across groups without significant differences between AAT groups and the placebo (*p* = 0.677 and *p* = 0.822 (AAT-60 mg/kg and AAT-120 mg/kg, respectively)). In all dosage groups, the decrease from baseline C-peptide AUC reached statistical significance: the placebo group declined by 0.34 pmol/mL (*p* = 0.008 from group baseline), the AAT-60 mg/kg group by 0.55 pmol/mL (*p* = 0.001), and the AAT 120-mg/kg group by 0.29 pmol/mL (*p* = 0.047).

**Figure 3:**
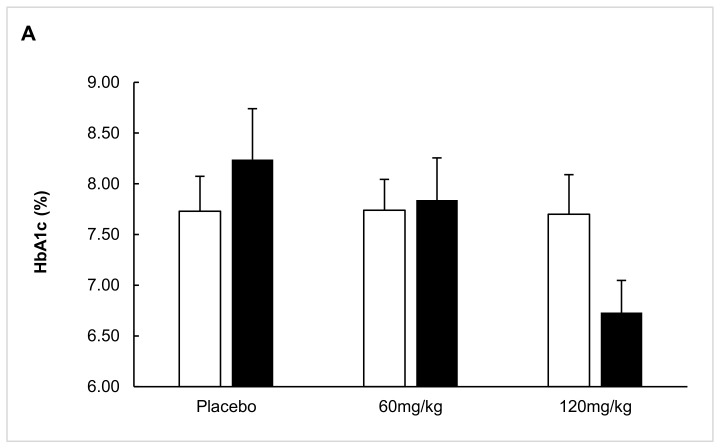

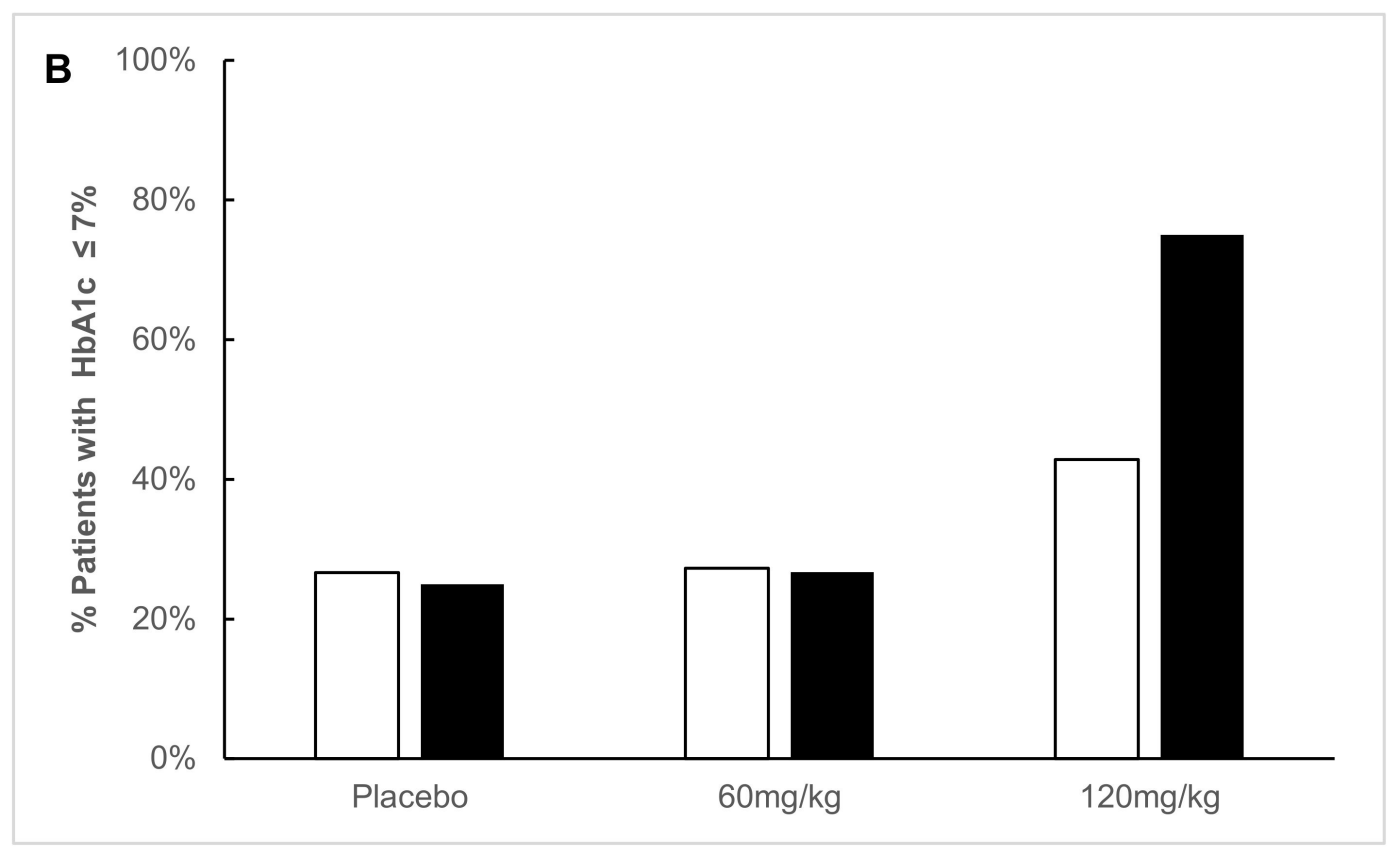
Glycemic control at the study’s end (52 weeks) stratified by treatment group. (**A**) Mean HbA1c levels of ITT versus 12–18-year subgroup. White bars = ITT analysis, black bars = 12–18-year subgroup. (**B**) Percentage of patients with HbA1c ≤ 7%, ITT versus 12–18-year subgroup. White bars = ITT analysis, black bars = 12–18-year subgroup.

A comparison of glycemic control parameters between groups upon study completion is presented in Figure 3. Mean HbA1c levels of 7.7% and the percentage of participants attaining HbA1c ≤ 7.0% did not differ between intervention groups. The daily insulin dose after 1 year was similar between treatment groups (mean 0.63 U/kg/d, *p* = 0.337).

### 2.2. Predefined Subgroup Analysis

Within the predefined subgroup of participants aged 12–18 years old (*n* = 35), week-52 C-peptide AUC levels displayed a nonsignificant tendency toward higher values in the AAT-120 mg/kg group compared to the placebo group (0.90 ± 0.23 pmol/mL vs. 0.48 ± 0.14 pmol/mL, *p* = 0.170). The C-peptide AUC in the AAT-60 mg/kg group was similar to the placebo (0.45 ± 0.06 pmol/mL, *p* = 0.866). Mean HbA1c levels were 8.2 ± 1.4%, 7.8 ± 1.6%, and 6.7 ± 0.9% (placebo, AAT-60 mg/kg, and AAT-120 mg/kg, respectively; *p* = 0.078 for AAT-120 mg/kg vs. placebo). HbA1c ≤ 7% was attained by a significantly higher percentage of patients in the AAT-120 mg/kg group compared to the placebo and AAT-60 mg/kg groups (75% vs. 25% and 27%, respectively; *p* = 0.050). The mean daily insulin dose tended to be lower in the AAT-120 mg/kg group on average compared to the placebo and AAT-60 mg/kg groups, albeit without reaching statistical significance (0.52 ± 0.10 U/kg vs. 0.68 ± 0.08 U/kg and 0.66 ± 0.09 U/kg, respectively; *p* = 0.209 for AAT-120 mg/kg vs. placebo).

### 2.3. Responders’ Analysis

The percentage of patients that maintained at least 95% of baseline C-peptide AUC was 6% in the placebo group and 19% and 16% in the AAT-60 mg/kg and AAT-120 mg/kg groups, respectively (*p* = 0.6). Meanwhile, in the 12–18-year subgroup, maintenance of a 95% baseline C-peptide reached 0%, 14%, and 29% (placebo, 60-AAT mg/kg, and 120-AAT mg/kg, respectively). The difference within the 12–18-year subgroup, nonetheless, did not reach statistical significance (*p* = 0.077, AAT-120 mg/kg vs. placebo).

### 2.4. Exploratory Analysis

A serum cytokine analysis was performed in order to explore the immune aspect of treatment outcomes. In all samples, IL-1β, IL-2, and IL-4 were below the lower limit of assay detection. However, changes from baseline cytokine levels were detectable in the case of IFNγ, TNFα, IL-1Ra, and IL-10 (Table A1). Changes in IL-10 levels were minimal and insignificant across treatment groups. Changes in IL-1Ra and TNFα displayed a trend toward a relative decline from baseline in the AAT-120 mg/kg group, albeit without reaching statistical significance.

### 2.5. Post Hoc Parametric Multivariate Analysis

In the predetermined analysis according to age group (8–11, 12–18, and 19–25 years old), a post hoc parametric multivariate analysis was carried out to evaluate the baseline characteristics that could have affected the treatment outcomes. The effect on β-cell function was evaluated as the relative change of C-peptide AUC from baseline to week 52 using a logarithmic transformation of AUC values. Mixed-model repeated measures with unstructured covariance matrices were used to fit the models, with the following baseline parameters incorporated: age, C-peptide AUC, HbA1c, BMI, time from type 1 diabetes diagnosis, and sex. An initial analysis indicated that the time from diagnosis, as well as sex, had no significant effect on C-peptide AUC over time, while baseline HbA1c, BMI, and C-peptide AUC had significant effects on outcomes (*p* = 0.0302, *p* = 0.006, and *p* < 0.0001, respectively). The effect of age ≥12 years had a borderline significance (*p* = 0.0545). Since when the sample size is small for a model using many parameters, this affects stability and may cause estimation problems, the analysis was carried out excluding the covariates that did not show a significant interaction with treatment. The effect of age ≥12 years was still found to be borderline (*p* = 0.073).

### 2.6. Safety Parameters

Adverse events classified by system organ class (MedDRA) and stratified by intervention group are presented in Table 2.

The infusions were tolerated well, with a similar safety profile in the AAT and placebo groups. The majority of adverse events were of mild or moderate intensity. Serious adverse events were reported in three patients allocated to AAT treatment: One patient in the AAT-60 mg/kg group was hospitalized due to mild DKA. One patient in the AAT-60 mg/kg treatment group had an allergic reaction during the fourth infusion, the study drug was withdrawn, and the patient fully recovered. One patient in the AAT-120 mg/kg group had a skin lesion removed during the surveillance period (after receiving 16 infusions), and pathological findings were consistent with fully excised superficial spreading melanoma Clark’s level II: this event was not likely related to the study drug, and the patient completed all serial AAT infusions per protocol. Three patients withdrew from the study due to adverse events: one patient in the placebo group withdrew after three infusions due to an anxiety response, which was resolved thereafter; the previously mentioned patient in the AAT-60 mg/kg group with an allergic reaction during the fourth infusion withdrew; and another patient in the AAT-60 mg/kg group with a mild infusion-related allergic reaction during the 17th infusion withdrew. No episodes of severe hypoglycemia were reported, and none of the participants had evidence of microvascular complications.

## 3. Discussion

In this double-blind, randomized, placebo-controlled, multicenter intervention trial, we could not demonstrate that AAT (Glassia^®^) had a beneficial effect on cell preservation and glycemic control in young patients with recent onset type 1 diabetes. However, in the absence of an adequate sample size to attain sufficient power, these findings should be interpreted with caution.

A possible positive effect on β-cell preservation was shown in adolescents (12–18 years old) who received the higher dosage of AAT. After 52 weeks, C-peptide AUC levels in the AAT-120 mg/kg adolescent group remained relatively stable in contrast to the decline observed in the placebo and AAT-60 mg/kg groups. In addition, the frequency of responders with at least 95% β-cell function reserve was 29% in the AAT-120 group and nil in the placebo. Glycemic control, as expressed by mean HbA1c levels, was improved in AAT-120 mg/kg-treated adolescents compared to placebo- and AAT-60 mg/kg-treated adolescents. In addition, a higher percentage of patients attained treatment targets of HbA1c ≤ 7% without higher daily doses of insulin. Of note is that these effects were demonstrated in adolescents and not in the overall intention-to-treat cohort.

This study brings us one step forward toward tailoring an immune-based intervention in an individualized manner. Today, it is well accepted that the emergence of type 1 diabetes at different age groups is also reflected in distinct epidemiology, pathophysiology, and response to therapy patterns [21,22]. Heterogeneity in β-cell recovery potential between different age groups has been suggested [21,22]. This phenomenon highlights the therapeutic diversity among patients. Therefore, it is plausible that specific age groups respond differently to a single medication [21,22]. Here, <12-year-old children were found to be poor responders to AAT, possibly due to the faster progression of type 1 in this age group [21,22] or a unique autoimmune pathogenesis. Our findings should be interpreted with caution, since the study population recruited was smaller than the calculated sample size, thereby reducing the statistical power from 80% to 57%. Still, further studies with AAT intervention should focus on this 12–18-years age group.

Exposure to immunotherapy during critical stages of childhood and adolescence raises concerns regarding the propensity to develop infection, autoimmune comorbidities, and malignancy. It is reassuring that multiple intravenous doses of AAT were found to be safe and well tolerated in children and adolescents with type 1 diabetes, independently of the tested dose range. Previous reports with AAT in recent-onset type 1 diabetes included smaller study populations with a wide age variability, doses up to 90 mg/kg/dose, and no placebo group [17,18,19,20]. Higher doses of AAT (>120 mg/kg per dose) have been explored both in preclinical and clinical studies [23], with no evidence for dose-dependency beyond 120 mg/kg per dose. In 2018, we reported on periodic AAT treatment (up to 36 infusions) in a cohort as being safe and well tolerated during a surveillance period of 5 years [20]. The present study adds to the cumulative evidence of its safety profile, as the majority of adverse events were of mild or moderate intensity. Neither severity nor the prevalence of side effects was related to AAT dosage or to age. Taken together, AAT interventions in type 1 diabetes have demonstrated a high safety profile; however, efficacy has yet to be determined.

Exploration of the immunomodulatory effects of AAT on β-cell apoptosis in type 1 diabetes is challenging. Changes in the serum levels of several cytokines were determined to have inconclusive results due to the small study sample, except for a trend in IFNγ levels (declining in the AAT-120 mg/kg group and rising in the placebo group). Unfortunately, our findings do not offer new insight into the immunological mechanisms related to AAT.

The strengths of the present study include the placebo-controlled and randomized design of the trial, compared to previous studies with AAT [17,18,19,20]. Additionally, the assessment of functional β-cell mass (C-peptide secretion during MMTT) to derive objective outcome measures represents a more direct probing of the course of disease at the β-cell level (compared to HbA1c). The limitations of this study include a lack of pharmacodynamic assays to determine the optimal dosage for achieving a therapeutic effect and a relatively small number of participants in each group.

## 4. Research Design and Methods

In this 52 weeks, phase 2, double-blind, randomized, placebo-controlled, multicenter intervention trial, AAT (Glassia^®^) was administered to patients with recent-onset T1D (ClinicalTrials.gov; NCT02005848). The study was conducted in four pediatric diabetes centers in Israel (the Assaf Harofeh Medical Center, Rambam Medical Center, Schneider Children’s Medical Center, and Soroka University Medical Center) from April 2014 to February 2017. The study was approved by the Ethics Review Board of each center and by the Israeli Ministry of Health and was conducted in accordance with the Declaration of Helsinki and the International Conference on Harmonization of Good Clinical Practice (ClinicalTrials.gov; NCT02005848). Prior to participation, informed written consent was obtained from adult participants and parents of minors; additionally, participants 12 years and older signed an assent form.

### 4.1. Participants

Seventy patients with recent-onset T1D were selected according to the following criteria: age 8–25 years, within 100 days from diagnosis, seropositive for diabetes-related autoantibodies [antibodies to glutamic acid decarboxylase (anti-GAD) or anti-islet], and stimulated C-peptide levels ≥0.2 pmol/mL. Excluded from the study were patients with immunoglobulin A (IgA) deficiency; recent treatment with live vaccination (within 1 month); corticosteroid treatment (within 2 months); the use of immunosuppressive or cytostatic agents (within 6 months); a history of severe immediate hypersensitivity reactions to plasma products and evidence of chronic and/or acute hepatitis; human immunodeficiency virus infection or parvovirus B19 infection; or significant abnormalities in hematology, blood chemistry, urinalysis, or electrocardiogram (ECG).

### 4.2. Randomization and Study Intervention

Patients who fulfilled all of the inclusion and exclusion criteria for the study were requested to return within 4 weeks of screening to participate in the treatment phase of the study. Eligible patients were randomized in a 1:1:1 ratio to receive either intravenous (IV) slow-drip AAT-60 mg/kg body weight, IV AAT-120 mg/kg body weight, or a matching placebo. A computer-generated random allocation sequence was prepared by the contract research organization (PAREXEL International GmbH, Berlin, Germany) in blocks of six (later amended to three) participants. Randomization codes were generated and stored under secure and blinded conditions: all study personnel and participants were masked to treatment allocation. Investigational medicinal product (IMP) was supplied to the pharmacy in each medical center by Kamada, Ltd. The IMP, Glassia^®^ (Kamada, Ltd., Rehovot, Israel), contained 2% active AAT in a phosphate-buffered saline solution, supplied in sterile, single-use glass vials containing 50 mL of ready-to-use solution. The placebo material was comprised of the nonactive ingredients of the preparation (NaCl in phosphate-buffered solution) and was supplied in identical packaging to that of the active drug. The doses were administered IV at a rate of up to 0.2 mL/kg/min using an appropriate infusion set for plasma products and incorporating a filter of 5 microns.

**Figure 4:**
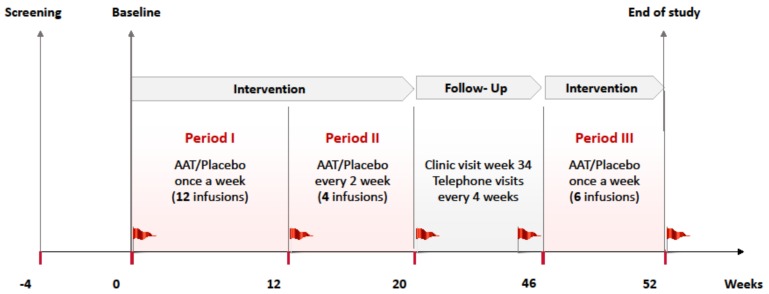
The study timeline: 22 infusions were administered over a period of 52 weeks. The first period consisted of 12 alpha-1 antitrypsin (AAT) or placebo infusions administered once per week. The second period consisted of 4 infusions administered every other week. There was a 26-week surveillance period followed by a third intervention period with 6 consecutive infusions. The red flags represent 2 h mixed-meal tolerance testing that was performed 5 times during the study. During the surveillance period, telephone visits were conducted every 4 weeks, and a clinic visit was done with a mixed-meal tolerance test (MMTT).

The 52-week study protocol was comprised of three intervention periods (up to 22 infusions)—period 1 (once per week for 12 consecutive weeks (12 infusions)), period 2 (once every 2 weeks for 8 weeks (4 infusions)), and period 3 (once per week for 6 weeks (6 infusions))—and a follow-up period, with serial assessment using a mixed-meal tolerance test (MMTT) at prespecified time points (Figure 4).

### 4.3. Laboratory Measurements

Blood samples were drawn according to protocol and sent to a central laboratory (Synlab Umweltinstitut GmbH, Berlin, Germany). A Siemens Healthcare Advia 2120i analyzer was used for the hematology panel, based on flow cytometry, hydrodynamic focusing, and the measurement of light absorbance of EDTA-collected whole blood. After separation of the serum, a biochemistry panel was performed using a chemiluminescent microparticle immunoassay on an Abbott ARCHITECT i2000 SR, and an AU 680 Beckman Coulter was used (based on photometry). Urinalysis was performed using a CLINITEK 500 Bayer HealthCare analyzer (based on reflectance spectrophotometry). Glycated hemoglobin (HbA1c) levels were determined by high-pressure liquid chromatography on a D-10 (BioRad). The MMTT was performed through ingestion of a carbohydrate-rich standardized liquid meal (Ensure^®^, Formula, Abbott Laboratories, Abbott Park, Il, USA) at 6 mL/kg/dose, and blood was drawn for C-peptide levels at times 0, 15, 30, 60, 90, and 120 min postingestion. The C-peptide response to the MMTT was expressed as basal, peak, and area under the curve (AUC). Serum C-peptide levels were determined using a chemiluminescent microparticle immunoassay on an ARCHITECT i2000 SR Abbott analyzer with a sensitivity of 0.027 pmol/mL. The analyzer was calibrated according to the international WHO international reference reagent for C-peptides of human insulin immunoassays. The inter- and intra-assay median coefficient of variation (CV) was set at 10%. Anti-AAT antibodies were assessed by Charles River Laboratories (Montreal, Canada) using a validated and qualitative bridging electrochemiluminescence immunoassay. Anti-GAD and anti-islet antibodies were determined at Euroimmun, Medizinische Labordiagnostika AG (Lübeck, Germany). Anti-GAD antibodies were tested by ELISA, and anti-islet antibodies were tested through an indirect immunofluorescence test. Serum levels of the following cytokines were tested at the end of the study (serum aliquots were kept frozen at −20 °C)—IL-1β, IL-2, IL-4, IL-10, IL-1Ra, interferon gamma (IFNγ), and TNFα—using Meso Scale Discovery (MSD) multispot assay system kits (V-Plex, Meso Scale Discovery A Division of Meso Scale Diagnostics, LLC, Rockville, MD, USA). Serum samples were handled and stored according to the manufacturer’s instructions. The lower limits of quantification were 2.14 pg/mL for IL-1β, 0.89 pg/mL for IL-2, 0.45 pg/mL for IL-4, 0.68 pg/mL for IL-10, 7.8 pg/mL for IL-1Ra, 7.47 pg/mL for IFNγ, and 0.69 pg/mL for TNFα.

### 4.4. Outcomes

The primary outcome was the level of β-cell function at 52 weeks from baseline, as determined by AUC-stimulated C-peptide. Secondary outcome measures included glycemic control (as expressed by HbA1c levels), insulin dosage, hypoglycemic events, and safety parameters (frequency and severity of clinical adverse events and physical, hematologic, and biochemical abnormalities). Exploratory outcomes included the percentages and characteristics of “responder” patients who maintained baseline levels of stimulated C-peptide as well as changes in serum levels of pro- and anti-inflammatory cytokines 52 weeks from baseline.

### 4.5. Sample Size, Data Management and Statistical Analysis

The sample size was calculated for the primary outcome measure on the basis of the decline in stimulated C-peptide AUC observed in a previous clinical study [19]. The calculation was based on demonstrating a difference of 21% in the primary endpoint between each of the treatment groups and the placebo group, with an α of 0.05 and a power (1-β) of 80%. The total sample size was estimated at 192 patients, with 64 per group. The study protocol was later amended due to an unexpectedly low enrollment rate to a total sample size of up to 75 patients, with up to 25 in each group. The change in design resulted in a decrease in the statistical power to approximately 57% for pooled AAT groups versus the placebo, assuming a 12% withdrawal to a final sample size of 66 patients.

All data were double-entered into an electronic data management system (PAREXEL, Version Y; PAREXEL International GmbH) and exported to the study database.

Descriptive statistics (the number of observations (*n*), mean, standard deviation (SD), arithmetic coefficient of variation percentage (CV% (if appropriate)), minimum, median, percentiles (25 and 75), maximum, and interquartile range (IQR)) were calculated for each quantitative variable. Categorical data were summarized by number of observations (*n*) and absolute and relative frequency. All data were analyzed as observed, with no imputation schemes for other missing values, except for a sensitivity analysis of the primary efficacy endpoint, where the “multiple imputation assuming missing-at-random and analysis” approach described by Mogg and Mehrotra [24] was applied using an adjustment to the methodology to accommodate the Wilcoxon test. Wilcoxon rank sum, Wilcoxon signed-rank, Kruskal–Wallis, or Fisher’s exact tests, as appropriate, were applied in order to test for a difference in the primary and secondary efficacy endpoints for each dose group compared to the placebo group. Student’s *t*-test (unpaired), Kruskal–Wallis, or Fisher’s exact tests were applied to the exploratory endpoints.

The following datasets were used for the analysis and presentation of the study data:

Intention-to-Treat Population (ITT): The ITT population was defined as all randomized patients who received at least one dose of study drug (AAT or placebo). Patients were included in the analysis as randomized. ITT population was analyzed for efficacy outcomes;

Age Subgroup Analysis: Patients were categorized according to their age at baseline, in 3 sets: children 8–11 years old, adolescents 12–18 years old, and young adults 19–25 years old. The subgroup analysis by age was carried out as a secondary analysis of efficacy on the ITT population;

Responders’ analysis: A post hoc analysis was carried out in which patients were categorized as “responders” and “nonresponders”. A “responder” was defined as having a postbaseline value that was at least 95% of the baseline value of C-peptide, an amount equal to one-half the interassay variation of the C-peptide assay (10%) [25].

All statistical analyses were performed using SAS^®^ (SAS Institute, Inc., Cary, NC, USA, Version 9.2 and 9.4). A two-sided *p*-value ≤ 0.05 was considered to be statistically significant.

## 5. Conclusions

AAT intervention comprises an immune-based intervention aimed at the preservation of residual β-cell function in recent-onset type 1 diabetes patients, with the advantage of a favorable safety profile. Our findings warrant further studies in a larger cohort to determine the impact of AAT on β-cell preservation and to individualize therapy according to the potential of response to this immune intervention.

## Figures and Tables

**Table 1:** Characteristics of patients upon randomization.

Variable	Placebo (*n* = 20)	AAT-60 mg/kg (*n* = 26)	AAT-120 mg/kg (*n* = 23)
Male sex	12 (60.0)	12 (46.1)	13 (56.5)
Age (years)	14 (4.8)	12.8 (3.3)	12.7 (4.3)
Children (8–11 years)	7 (5.0)	8 (30.8)	12 (52.2)
Adolescents (12–18 years)	9 (45.0)	17 (65.4)	9 (39.1)
Young adults (19–25 years)	4 (20.0)	1 (3.8)	2 (8.7)
BMI (kg/m^2^)	19.5 (3.8)	19.2 (2.8)	19.3 (4.0)
BMI-SDS (8–19 years)	0.10 (1.39)	0.22 (0.91)	0.22 (1.15)
Time since diagnosis (days)	67.1 (23.7)	71.7 (26.2)	75.1 (28.6)
Diabetic ketoacidosis at diagnosis	11 (55.0)	9 (34.6)	11 (47.8)
HbA1c (%)	8.5 (1.5)	8.5 (2.2)	8.7 (1.8)
HbA1c (mmol/mol)	69 (16)	69 (23)	72 (19)
C-peptide, fasting (pmol/mL)	0.24 (0.14)	0.20 (0.11)	0.22 (0.13)
C-peptide, stimulated (pmol/mL)	0.68 (0.39)	0.63 (0.35)	0.59 (0.34)
Glutamic acid decarboxylase autoantibodies	19 (95)	24 (92)	22 (96)
Islet-cell autoantibodies	9 (45)	15 (58)	12 (52)
Positive for two autoantibodies	8 (40)	14 (54)	11 (48)

Note: data are mean (SD), *n* (%).

**Table 2:** Adverse events stratified by intervention group.

System Organ Class Preferred Term	Placebo		AAT-60 mg/kg		AAT-120 mg/kg	
	No. Patients	No. Events	No. Patients	No. Events	No. Patients	No. Events
Infections: nasopharyngitis, gastroenteritis, viral infections, upper respiratory tract infections	17	51	20	57	21	63
Nervous system: headaches and dizziness	10	78	20	91	18	117
Gastrointestinal: abdominal pain, diarrhea, vomiting, dyspepsia	12	56	16	59	14	91
General disorders and administration site conditions	10	43	16	50	12	43
Respiratory, thoracic, and mediastinal	7	11	15	18	9	33
Musculoskeletal and connective tissue	5	7	7	11	6	13
Skin and subcutaneous tissue	3	8	7	7	5	11
Eye disorders: blurred vision, eye pain, or swelling	3	4	2	3	4	4
Injury or poisoning	5	7	4	6	4	5
Renal and urinary tract: dysuria, ketonuria, microalbuminuria	0	0	0	0	3	3
Reproductive system: dysmenorrhea, menstrual discomfort	2	2	2	4	2	31
Metabolism and nutrition	4	5	5	9	2	2
Blood and lymphatic system	2	2	1	1	2	2
Psychiatric disorders: anxiety	2	3	1	1	2	2
Endocrine disorders: thyroiditis, hypothyroidism	0	0	0	0	2	2

Note: The analysis of adverse events was carried out on the study population, including all reported adverse events, regardless of an assessment of causality from the study medication.

## Appendix A

**Table A1:** Change in cytokine serum levels.

Treatment:	Placebo		AAT-60 mg/kg		AAT-120 mg/kg	
Population:	ITT	12–18 years	ITT	12–18 years	ITT	12–18 years
**IL-1 Ra (pg/mL)**						
Mean (SD)	35.07 (344.94)	6.38 (451.79)	−42.11 (218.51)	7.85 (140.44)	−90.88 (161.02)	−169.29 (178.98)
Median (IQR)	−25.00 (205.75)	−69.50 (265.00)	6.00 (181.5)	31.00 (208.00)	−87.00 (208.00)	−162.00 (102.50)
*p*-value			0.47	0.99	0.22	0.34
**IL-10 (pg/mL)**						
Mean (SD)	0.14 (0.57)	0.20 (0.77)	−0.05 (0.18)	−0.02 (0.15)	0.03 (0.19)	0.06 (0.25)
Median (IQR)	0.09 (0.26)	0.15 (0.57)	−0.01 (0.17)	0.04 (0.19)	0.00 (0.22)	−0.06 (0.31)
*p*-value			0.25	0.45	0.51	0.65
**IFN** **γ (pg/mL)**						
Mean (SD)	13.50 (27.91)	16.85 (32.81)	−2.10 (8.98)	−3.09 (10.73)	−1.34 (3.40)	−1.13 (2.18)
Median (IQR)	0.50 (0.57)	0.50 (18.58)	−0.30 (4.8)	−0.30 (6.00)	−1.20 (4.63)	−1.70 (2.8)
*p*-value			0.06	0.13	0.07	0.17
**TNF** **α (pg/mL)**						
Mean (SD)	0.51 (1.26)	0.69 (1.59)	1.24 (4.07)	1.86 (4.79)	0.14 (0.94)	−0.07 (1.31)
Median (IQR)	0.40 (0.70)	0.40 (0.83)	0.40 (0.98)	0.41 (0.80)	0.30 (0.66)	0.10 (0.90)
*p*-value			0.47	0.43	0.37	0.33

Note: The analysis was carried out on the ITT population and the 12–18-years-old subgroup, and the changes in the cytokine levels were calculated from baseline (before the first administration of AAT or the placebo) to the end of the study 52 weeks later. SD = standard deviation; IQR = interquartile range; *t*-test *p*-value is the result of a two-sided, unpaired Student’s *t*-test of each AAT treatment groups versus the placebo, assuming an unequal variance in the various groups.
